# Perimetry of the Central Visual Field Using a Head-Mounted Open-Source Perimeter in Patients with Inherited Retinal Diseases

**DOI:** 10.3390/vision10010012

**Published:** 2026-02-14

**Authors:** Cord Huchzermeyer, Friedrich Kruse, Jan Kremers

**Affiliations:** 1 Department of Ophthalmology, University Hospital Erlangen, 91054 Erlangen, Germany; 2 Department of Ophthalmology, Friedrich-Alexander-Universität Erlangen-Nürnberg, 91054 Erlangen, Germany

**Keywords:** head-mounted perimetry, virtual reality, inherited retinal diseases

## Abstract

Head-mounted (“virtual reality”) perimeters (HMPs), based on standard consumer electronic hardware, are a cheaper alternative to standard automated perimetry. They have not been validated in patients with inherited retinal disease (IRDs), yet. We evaluated the Iowa-HMP in a first pilot study. It consists of a legacy smartphone, a headset, and freely available, open-source software. We used the 10-2 grid, the ZEST algorithm, and a background of 10 cd/m^2^ to measure central visual fields in one normal subject, and in patients with occult macular dystrophy (n = 2), Stargardt’s disease (n = 3) and retinitis pigmentosa (n = 6). Results were compared with those from an Octopus 900 perimeter. The typical patterns of visual field loss were clearly discernible, but head-mounted perimeters generally have a limited dynamic range. Within the dynamic range of the Iowa-HMP (14 to 30 dB Octopus sensitivity), the Limits of Agreement (Bland-Altman) were ±7.5 dB. The Iowa-HMP had a diagnostic sensitivity of 0.67 for detecting locations with low perimetric sensitivity (<14 dB in the Octopus perimetry) with a diagnostic specificity of 0.95. Although the Iowa-HMP cannot be directly compared to standard perimetry in IRDs, open software greatly facilitates research in this area.

## 1. Introduction

In inherited retinal diseases (IRDs, see Abbreviations), perimetry is an important tool that allows not only the identification of typical patterns of visual field loss but also the quantification of photoreceptor function by measuring decreased differential light sensitivity [1,2]. Standard automated perimetry (SAP) uses projection-based perimeters with white stimuli on a white background [3].

The first automated (computer-driven) static perimeters were developed in the 1970s: the Octopus 201 (Haag-Streit, Köniz, Switzerland) [4,5] and the Humphrey Field Analyser (Carl Zeiss Meditec, Inc., Dublin, CA, USA) [6,7]. These consist of a dome with diffuse background illumination, on which focal stimuli are projected as light increments. During the test, the patient places his head on a chin rest and fixates the center of the dome. The stimuli are modified according to the patients’ responses (a pressing of a button that is registered within a response window indicates that the luminance increase was seen) following predefined algorithms to effectively determine the light increment detection thresholds. With these devices, very strong light increments are possible because the stimuli are created with a xenon arc lamp or with modern, very bright LEDs and superimposed on the background. In addition, neutral density filters facilitate very small luminance increments, enabling a very large technical dynamic range [8].

Perimetry usually tests observers’ sensitivities to differential light increments [3]. Absolute scotomas are areas of the visual field where there is no light perception at all; relative scotomas allow detection of lights that are brighter than necessary for a normal observer. In these relative scotomas, the threshold denotes the light increment that is just detected by the observer [3]. Perimetric sensitivity is a logarithmic measure that relates the individual’s increment threshold to a reference, so that lower thresholds correspond to a higher perimetric sensitivity and vice versa. By convention, the largest increment ($\Delta L_{max}$) that can be presented by a given perimeter is chosen as the 0 dB reference luminance (see the supplement to [3]). Thus, the sensitivity dB-scales of different perimeters define different stimulus brightness levels as 0 dB sensitivity. In clinical practice, the implication of this is mitigated because the sensitivity deviation (or defect), that is the difference between the observed perimetric sensitivity and that of an age-matched or age-corrected normal population, is used instead of the perimetric sensitivity. Because the difference to the 0 dB reference level is present in both the observed and the normal values, it is eliminated from the equation. During the last decades, the background luminance settings ($L_{background}$) have converged across the perimeters, and low photopic settings of 10 cd/m^2^ are generally used (historically, the Octopus perimeters used 1.27 cd/m^2^) [4].

In contrast, head-mounted perimetry has emerged only recently as an interesting alternative, relying on commercially more widely available and, therefore, much cheaper components that are also used in consumer electronics [9]. They are either based on smartphones and a headset or on full-fledged virtual-reality platforms. This technical development allows a more widespread application in underserved communities and telemedical settings [10]. However, background and stimuli are presented on OLED or LCD displays which have a much smaller maximal luminance and often provide only 255 equal luminance steps, owing to the RGB additive color model and thus to a limited resolution [11]. This limits direct emulation of projection-based perimeters [11,12]. In a number of devices, lower background settings in the mesopic range are used in order to enable larger luminance increments.

When perimetric sensitivity is systematically mapped across the visual field, the resulting pattern is often interpreted by pattern recognition (“gestalt” perception), but this might be misleading [13]. Therefore, a quantitative analysis of SAP results is necessary [3]. In inherited retinal diseases (IRDs), perimetry is used to identify characteristic patterns of visual field loss, including central scotoma [14], ring scotomas or concentric constriction [15,16]. Perimetry is used as a functional screening test for toxic maculopathy in patients under (hydroxy-)chloroquine therapy [17]. In addition to supporting the clinical diagnosis, visual field tests support clinicians in counseling the patient on a range of issues like driving or occupational safety. Quantification of visual field losses is also important in clinical research. They are an integral part of phenotype-genotype correlations [18], and a number of perimetric techniques are discussed as endpoints in clinical trials of gene- and cell-based therapies of inherited retinal diseases [2,15,19]. However, new techniques, including fundus-controlled perimetry [20], dark-adapted perimetry [15], chromatic perimetry [2], and volumetric analysis of the hill of vision [21] are frequently advocated to overcome limitations of SAP for such endpoints.

The Iowa head-mounted perimeter is a cheap open source perimeter for research purposes [22]. It is based on a legacy smartphone mounted in a virtual reality headset. The freely available Phone HMD app on the smartphone is driven by the (also) freely-available OpiApp via a local WiFi network. In contrast to some commercially available head-mounted perimeters, the data are saved locally on the desktop computer and not on an external server, and can be exported completely. This software is provided by the Open Perimetry Initiative (OPI) [23,24]. The purpose of the present study was to provide a first proof-of-principle for using head-mounted perimetry in inherited retinal diseases with an open-source device.

## 2. Materials and Methods

**Figure 1:**
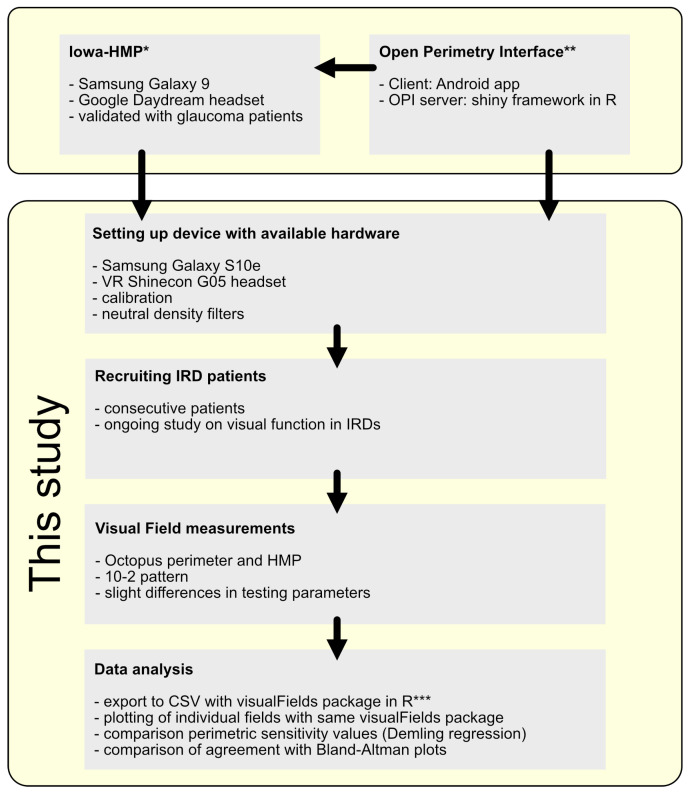
Flow chart giving an overview over our study and its relationship to previous work by other groups (* Heinzman 2023 [22], ** Turpin 2012 [24], *** Marín-Franch 2013 [29]).

An overview of our study and its relationship to previous work by other groups is shown in Figure 1.

### 2.1. Patients

We examined 11 patients and one normal subject with ages between 24 and 73 years (median: 58 years, IQR: 23 years). The demographic and clinical characteristics of our study cohort are shown in Table 1. The patients had participated in earlier studies [25,26,27] and were seen yearly in our retina clinic. All perimetric measurements reported here were performed in 2025. All cases of ABCA4-associated disease [26] and RP1L1-associated occult macular dystrophy [27] were genetically confirmed. In contrast, diagnosis of RP was made clinically [25]. However, genetic results were available for most patients, and two women with clinically manifest heterozygous RPGR mutations were included [28]. All patients had been examined at least three times with the Octopus perimeter (usually G1 or M pattern) but not with the VR perimeter prior to this study.

This study adhered to the tenets of the declaration of Helsinki and was approved by the Ethics committee of the Medical Faculty of the Friedrich-Alexander University Erlangen-Nürnberg (300-17B). Patients gave written informed consent prior to participation.

### 2.2. Device 1: Iowa Head-Mounted Perimeter

The device consisted of a commercially available smartphone, a headset, and a personal computer which drives the smartphone via a client-server-infrastructure in a local WiFi network. The total price of the smartphone, the headset, the neutral density filter, and a USB WiFi adapter was below 300 Euro.

#### 2.2.1. Smartphone and Headset

**Figure 2:**
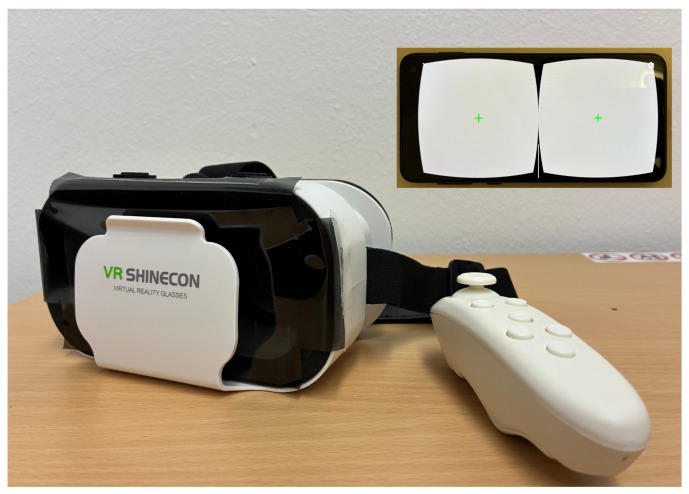
Headset equipped with a 0.6 neutral density filter foil. A Samsung galaxy S10e is inserted into this headset for perimetry. The inset shows the smartphone display with the perimetry app running. The total price for the device (excluding the personal computer running the software) was below 300 Euro.

We used a Samsung Galaxy S10e (SM-6970U1) with a 147 mm (5.8 in) screen (2280 × 1080 pixels) and an Android 12 operating system together with a VR Shinecon headset. The refresh rate is 60 Hz. An optical neutral density (ND) filter (0.6 OD units) was placed between the smartphone screen and the headset (see Figure 2). The opiPhoneHMD app (opiPhoneHMP-release.apk) was downloaded from www.optocom.es (accessed on 16 May 2025) and installed on the smartphone.

The headset is 4.5 cm from the pupil, but the headset lenses magnify the image by a factor of 2–3 in order to fill the field of view as much as possible ($100^{\circ }$). According to the user manual the viewing effect is equivalent to watching a 26.7 m (1050 in) screen at a distance of 3 m. The resulting optical distortion is corrected by the app. Since no dedicated Google Daydream QR code (which allows the VR app to correct for lens distortion) was provided by the manufacturer together with the headset (VR Shinecon G05), we used a QR code published online for this headset (https://www.shinecon.com/news/vr-shinecon-qr-codes-g10-g04-g05-g06-g07-serie-85032053.html, accessed on 27 January 2025).

#### 2.2.2. Software

A conventional personal computer, running on Windows 10, was used to drive the visual field test with the OPI app for R (version 4.5.1, “Great Square Root”). It was necessary to install the older version 2.11.2 instead of the current version 3.x of the OPI package. The Smartphone was calibrated using a Minolta LS-110 Luminance Meter from a distance of >1 m.

#### 2.2.3. Settings

The background was set to 10 cd/m^2^ (as seen through the ND filter; i.e., the screen was set to 39.8 cd/m^2^ on the phone). This allowed minimal luminance increments of 0.13 cd/m^2^, and maximal luminance increments of 12.76 cd/m^2^. The program used the 10-2 pattern, a Goldmann III stimulus size (0.43deg visual angle), and 100 ms stimulus duration.

### 2.3. Device 2: Octopus 900 Projection-Based Perimeter

Standard automated perimetry was performed on the Octopus 900 (Haag-Streit, Köniz, Switzerland) using the Haag-Streit EyeSuite Software (2.3.1). We also used the 10-2 pattern, a Goldmann III stimulus size (0.43deg visual angle), and 100 ms stimulus duration. Contrary to current practice and to the VR perimetry settings, a background luminance of 1.27 cd/m^2^ (4 asb), and a maximum ΔL of 318 cd/m^2^ (1000 asb) was used. The parameters used by the two devices are compared in Table 2.

### 2.4. Data Analysis

We analyzed only differential light sensitivity (i.e., the ability to detect small light increments, measured in decibel [dB]), because no age-related reference values were available for the head-mounted perimeter. For simplicity, differential light sensitivity will be called perimetric sensitivity throughout the manuscript. Perimetric sensitivity should not be confused with diagnostic sensitivity [%] (the ability of a test to detect the presence or absence of a condition), which is also used in the manuscript (see below).

The perimetric sensitivity is calculated as

(1) $$perimetric\;sensitivity\;\left[\mathrm{dB}\right]=10\;log\left(\frac{\Delta L_{max}}{\Delta L_{threshold}}\right)\;.$$

Thus, a perimetric sensitivity of 0 dB is defined as the ability to just detect the most luminant stimulus that can be presented during the examination. This results in different offsets of the dB-scale in the two devices. Also note that, by convention, perimetric sensitivity does not depend on the background luminance.

Throughout the manuscript, we compare uncorrected sensitivity values, and not “equivalent sensitivity” values (where the dB scales are corrected for differences in $\Delta L_{max}$), except for the visual field printouts. These were plotted with the vfplot() function of the visualFields library [29] for the statistical programming language R. Due to the vastly different dynamic ranges and the resulting floor effects, it is not reasonable to compare global indices like mean sensitivity (MS).

For comparison of perimetric sensitivity values between both modalities, Deming regression was used, because both measures have a measurement error [30].

Bland-Altman plots were created to compare perimetric sensitivities while censoring locations where sensitivities were smaller than 14 dB in the Octopus perimetry. Both eyes of each subject were included in the analyses, because the differences within visual fields (i.e., between locations with good sensitivity and with poor sensitivity) is much higher than inter-individual variability. No dedicated correction for the correlation between both eyes of each patient was applied, because we performed no formal hypothesis testing or inferential statistics. Analyses were performed for all 68 locations of each field (pointwise).

For Bland-Altman-plots, locations with Octopus sensitivities below 14 dB were censored. Instead, we used a contingency table and diagnostic sensitivity and specificity in order to evaluate the ability of the head-mounted perimeter to detect low perimetric sensitivities [dB] (<14 dB in the Octopus field).

Diagnostic sensitivity and specificity are calculated as follows:

(2) $$diagnostic\;sensitivity\;\left[\%\right]=\frac{true\;positives}{true\;positives+false\;negatives}\;.$$

(3) $$diagnostic\;specificity\;\left[\%\right]=\frac{true\;negatives}{true\;negative+false\;positive}\;.$$

## 3. Results

### 3.1. Relationship Between Perimetric Sensitivity Scales

**Figure 3:**
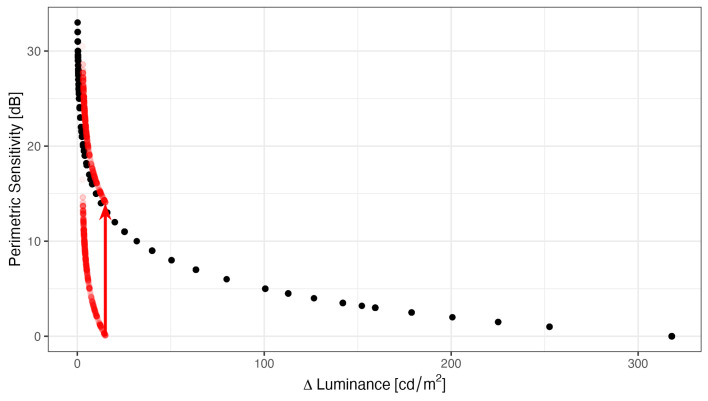
Stimulus luminances (ΔL) and the corresponding retinal sensitivities that were observed in our study population with the Octopus perimeter (black) and the HMP (transparent red points, darker red points are caused by overplotting of several points). There is a simple mathematical relationship between Luminance and Perimetric Sensitivity, which is elaborated in Equation (1). The HMP values are shifted upward by 14 dB (red arrow) to account for the different $L_{max}$ and slightly shifted to the right to avoid plotting over Octopus data. The purpose of this figure is to show the range of values observed with each device and to highlight why a very small luminance increment is necessary for 1 dB difference in perimetric sensitivity in areas with high sensitivity, whereas a large luminance increment is required in areas with low sensitivity.

Figure 3 shows the pointwise sensitivities and the corresponding threshold luminance increments that were observed with the two perimeters in our subjects. The plot illustrates the logarithmic relationship between luminance increments and perimetric sensitivities. By convention, perimetric sensitivities are quantified relative to the brightest stimulus the device can present, and therefore, 14 dB must be added to the HMP sensitivities, so that identical luminances correspond to identical sensitivities between the perimeters. The dynamic range of the Octopus (33 dB) is much larger than that of the Iowa HMP (16 dB).

Note that Figure 3 also illustrates that the Octopus is capable of measuring both (1) higher perimetric sensitivities (because of the higher luminance resolution) and (2) lower sensitivities (because it can produce more luminous stimuli). The inability of the head-mounted perimeter to measure high sensitivities, as observed in normal subjects in the fovea, because very small changes in luminance on the left hand side of the plot result in large changes in perimetric sensitivity (ceiling effects). The HMP cannot produce sufficiently small luminance increments.

On the other hand, the HMP cannot measure sensitivities below 14 dB Octopus equivalent perimetric sensitivity because it cannot produce more luminous stimuli (floor effects). Without the 0.6 ND filter, the dynamic range would extend more into the low perimetric sensitivity regions and floor effects would have been mitigated. However, the cost would have been larger ceiling effects, a flattened hill of vision and the inability to measure small perimetric sensitivity losses in the foveolar region [22].

**Figure 4:**
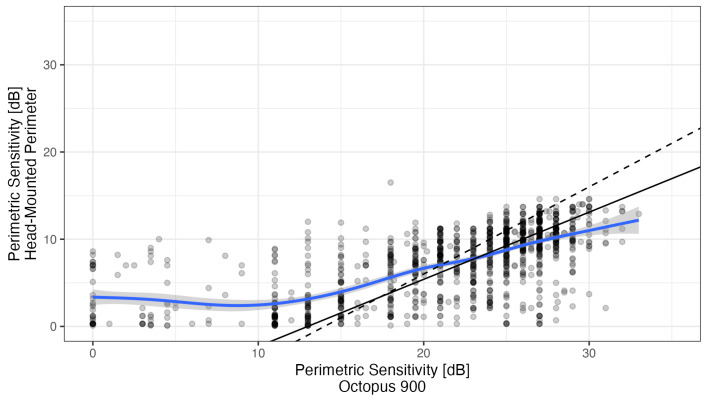
Relationship between the measured Octopus sensitivities and the corresponding head-mounted perimetry sensitivities. Points are black with a transparence, so that they appear gray. Darker black points are caused by overplotting of several transparent points. This allow understanding the fit of the lines through the denser areas. The blue line shows a spline fit, which highlights the floor effects below 14 dB sensitivity. The drawn black line shows the Deming regression line through the points with Octopus sensitivities larger than 14 dB and smaller than 30 dB (floor and ceiling effects expected in the HDM perimetry). The dashes line shows the line with a slope of 1 and an intercept of −14 dB that would be expected if both devices were equivalent.

Deming regression was used to characterize the relationship between pointwise HMP and Octopus sensitivities (see Figure 4, no correction for the different 0 dB setoff). Octopus sensitivities between below 14 dB and above 30 dB were censored from regression analysis, because ceiling and floor effects occur with the Iowa-HMP outside that range. Within this range, regression yielded a slope of 0.77 (95% confidence interval 0.70 to 0.84) with an intercept of −10.0 dB (95% CI: −11.8 to −8.3). This slope is smaller than the slope of 1 that would be expected if stimulus conditions, especially background luminance, had been identical in both perimeters. The spline fit (without censoring) suggested an even smaller slope of approximately 0.5. However, the deviation from the theoretical slope of 1 is small compared to the variability of perimetric measurements.

Therefore, we calculated HMP-measured “Octopus equivalent” sensitivities by simply adding 14 dB without correcting for slope for further comparison.

### 3.2. Agreement Between Measurements

**Figure 5:**
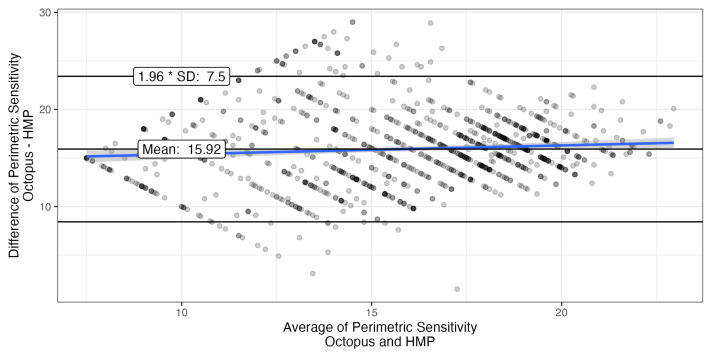
Bland-Altman plot showing the agreement between Octopus perimeter and head-mounted perimeter for locations with Octopus sensitivities >14 dB (transparent black points; single points appear gray; dark black points are caused by overplotting of several transparent points). Looking for agreement is pointless when sensitivities are outside the dynamic range of one device. For this analysis, HMP sensitivities below the dynamic range were either set to 0 or censored when Octopus sensitivities were also low. The systematic difference exceeds that predicted by the difference in offset by almost 2 dB, possibly due to the differences in background.

The Bland-Altman plot showed good agreement for locations with Octopus sensitivities > 14 dB with limits of agreement (LoA) of ±7.5 dB, and even better agreement for higher sensitivities (Figure 5). Only values were compared that were within the dynamic range of both devices. The systematic difference exceeds the 14 dB predicted by the difference in offset by almost 2 dB, possibly due to the differences in background.

Thus, in order to compare agreement of perimeters in the low perimetric sensitivity range, we created a contingency table (Table 3). The HMP’s diagnostic sensitivity (true positives divided by true positives + false negatives) to detect Octopus perimetric sensitivity values below 14 dB was 0.67 and diagnostic specificity was 0.95. This suggests that the HMP has a tendency toward higher sensitivities close to the lower limit of its dynamic range.

### 3.3. Patterns of Visual Field Loss

The “Dynamic” algorithm used by the Octopus perimeter had longer exam durations (4.3 min to 16.1 min; IQR: 4.7 min; median 7.7 min) compared with the ZIPPY algorithm of the HMP (2.1 min to 12.6 min; IQR: 2.4 min; median 6.1 min), despite a similar median number of presentations of the Octopus software (181 to 306; 130 to 531; IQR: 125; median: 215), compared with the HMP (77 to 424; IQR: 72; median: 222). This depends on testing strategies and is not inherent to the devices.

**Figure 6:**
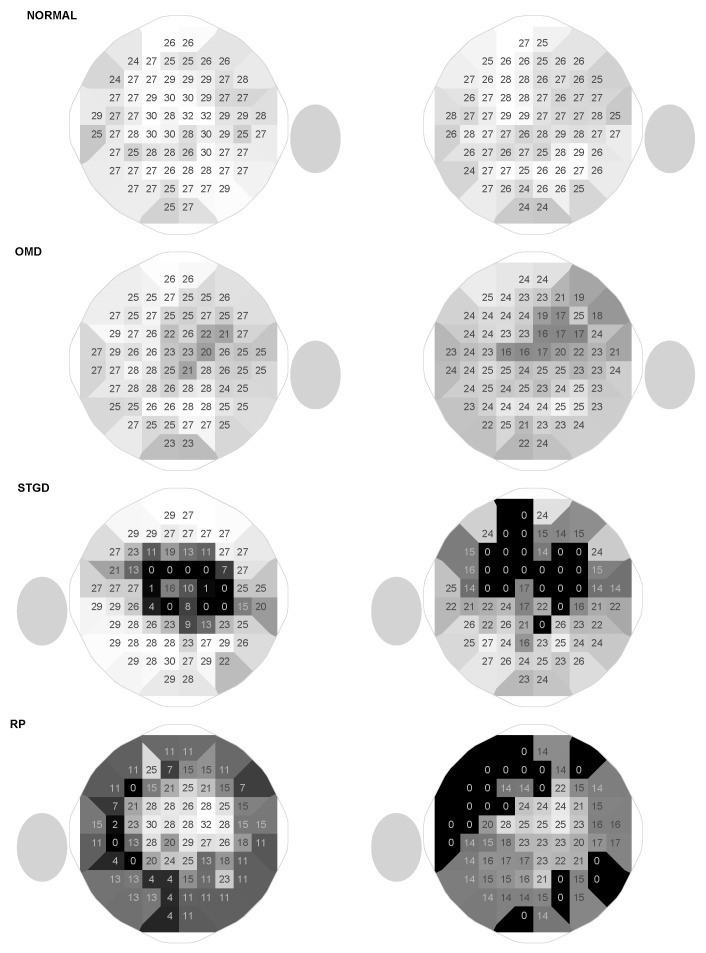
Comparison of characteristic fields of four subjects (normal, occult macular dystrophy, Stargard’s disease, and retitinitis pigmentosa) between the Octopus perimeter (left side) and the head-mounted perimeter (right side). The plots show the Octopus-equivalent sensitivities [dB] (14 dB added to HMP values, HMP values of 0 dB remain 0 dB) for each location in the 10-2 grid with additional grayscale coding of these values in the tessellation to allow perception of typical patterns. The gray ovals show the location of the physiological blind spot, which is outside the test field but allows identification of laterality (right eyes: blind spot is on the right side).

The overall patterns are similar with both devices, and the characteristic patterns of occult macular dystrophy (OMD), ABCA4-associated Bull’s-Eye-maculopathy and retinitis pigmentosa could be clearly appreciated (Figure 6) with the HMP. In the OMD subjects, the central perimetric sensitivity losses were more pronounced with the HMP. In the other two disease groups, there were transition zones with slightly decreased sensitivities, where losses were clearly overestimated with the HMP (for example the deep superior defects in the patient with ring Bull’s-Eye scotoma, see Figure 6).

## 4. Discussion

This pilot study shows that head-mounted perimeters can show typical visual field patterns in inherited retinal diseases, including decreases in central perimetric sensitivity cause by occult macular dystrophy, bull’s eye defects and central scotomas in Stargardt’s disease and concentric defects in retinitis pigmentosa. However, the pointwise comparison shows that HMP perimeters are not equivalent to projection-based systems. For this study, we used a low-budged, open-source head-mounted perimeter [22], which is based on the open-perimetry initiative (OPI) interface [23,24,29]. Open software allows modification of testing protocols and greatly facilitates research.

### 4.1. Differences in Measurements Between the HMP and the Projection-Based System

Although typical visual field loss patterns could be discerned in our study, measurements were not equivalent. Foremost, the HMP (like other HMP devices [11]) has a much smaller dynamic range than the Octopus perimeter, resulting in both floor and ceiling effects (see Figure 3). Therefore, in the normal subject in Figure 6, central sensitivities were lower. With a background luminance of 10 cd/m^2^, our device had a dynamic range of 16 dB, which means that the strongest stimulus is 40 times brighter than the dimmest stimulus. In contrast, the Octopus perimeter has a much larger dynamic range of 33 dB. On the other hand, the minimal luminance increments that can be achieved with head-mounted perimeters are too large to measure the very high sensitivities in the fovea. Because head-mounted perimeters are RGB displays, there are 255 equally spaced luminance increments between the smallest and the largest ΔL. This results in a flattened hill of vision centrally.

Following the study by Heinzman and coworkers [22], we used a neutral density (ND) filter to mitigate the ceiling effect at the cost of a more pronounced floor effect. The neutral density filter can be omitted when patients with low sensitivities are examined, but perimetry is more reliable in areas of high perimetric sensitivity, so that the effective dynamic range is likely improved by the ND filter [31]. The limited dynamic range is inherent to the underlying hardware and affects all HMPs, even though some of them have a larger dynamic range [11]. Technically, dynamic range can be expanded using software techniques like dithering or different hardware, for example 10 bit displays.

Some HMP devices drastically reduce background luminance in order to expand dynamic range [12,32], but this only mitigates floor and not ceiling effects. Furthermore, due to the different adaptive state of the retina, photoreceptor damage can have different functional consequences with different background luminances [8].

In our study, the background luminance was lower in the Octopus perimeter, because, inadvertently, a protocol with legacy settings was used throughout the study (these settings allow comparison with older measurements from Octopus 500 perimeters for a longitudinal study with very long follow-up [33]). In the perimeter with lower background luminance, the Weber contrast is increased for any given $\Delta L_{max}$. Therefore, systematically and constantly higher sensitivities would be expected for the Octopus perimeter in our study, had both perimeters operated in the Weber range (where perception depends on contrast rather than absolute luminances). Thus, the slope of the Deming regression confirms that perimeters do not operate in the Weber range, at least at 1.27 cd/m^2^ [4]. The 15.92 dB systematic difference in the Bland-Altman analysis exceeds the 14 dB predicted by the difference in $\Delta L_{max}$, but not by the amount predicted by the difference in Weber range. Again, such problems may affect all perimeters that use different background luminances. In several publications, very low background luminances were used in the head-mounted perimeters in order to increase the dynamic range [12,32]. On the other hand, these limitations also offers the opportunity to question old standards and new testing protocols could be developed in order to improve diagnostic power [8].

In contrast to the previous study by Heinzman et al. [22], we used the Eyesuite software provided by the manufacturer (Haag-Streit, Köniz, Switzerland) for driving the Octopus perimeter and not the Open Perimetry Interface used for the HMP. Thus, we could not use the same testing strategy. This might slightly limit agreement, but the effect is probably negligible compared to the factors mentioned above.

Given these differences, the limits of agreement in the Bland-Altman analyses (±7.5 dB) were quite good. This was also comparable to earlier comparisons of the Iowa head-mounted perimeter with the Octopus 900 in normal subjects (mean difference 0.4 dB, limits of agreement ±4.6 dB) and patients with glaucoma (mean difference 0.1 dB, limits of agreement ±8.9 dB).

### 4.2. First Use of Head-Mounted Perimeter in Patients with IRDs

To our knowledge, this is the first study that demonstrates the feasibility of perimetry measurements with HMPs in patients with IRDs. Typical disease patterns could be demonstrated despite limiting floor- and ceiling-defects.

Interestingly, central sensitivities of the patients with occult macular dystrophies were lower when measured with the head-mounted perimeter. Possibly, this could be explained by the differences in background luminance. The structure-function relationship is complex in retinal diseases and depends on the affected photoreceptor types and the retinal adaptation [8,34]. In the fovea, the parvocellular system is more dominant compared to the peripheral retina and it may be involved in the detection of perimetric stimuli [35], particularly for relatively long stimulus presentation times ≥100 ms as used for perimetry [36]. Finally, the area of complete summation (Ricco’s Area) is smaller in the central retina, so that perimetric stimuli are frequently larger than this area, which is not optimal for reproducibility of results [12].

On the other hand, darker backgrounds might be superior to the 10 cd/m^2^ used in SAP [2,37]. Furthermore, protocols where stimulus size is adjusted instead of luminance (size threshold perimetry) could be investigated for improving reliability of measurements in inherited retinal diseases [38]. Thus, the introduction of HMP might facilitate improvements of testing protocols, as long as researchers have the opportunity to experiment without limitations imposed by manufacturers who are interested in a rapid proof of equivalence with SAP [10].

### 4.3. Open-Source HMP

For the Iowa HMP, considerable patience and knowledge of the programming language R, networking and luminance measurements are necessary in order to set up the device, because it is based on open-source projects and because it is no longer fully supported by the maintainers. Legacy equipment (smartphone) and neutral density filters are needed for building the device [22]. Most importantly, it cannot be used for clinical diagnosis, because has neither a CE-mark nor approval as a medical device.

The advantages of the Iowa head-mounted perimeter are the low price, the experimental degrees of freedom concerning the test parameters, and the control over the collected data, which is saved locally and completely available for analysis. In our case the price was less than 300 Euro, excluding the desktop computer and the equipment for a local WiFi, which were already part of the Lab infrastructure. Many commercially available head-mounted-perimeters process data on a backend server (which can be in another country) [9].

Other smartphone-based visual field tests have been published [32,39,40], but the Iowa-HMP is the only one that is based on open-source software, where the full source code is available for research purposes [24]. Furthermore, this source code is developed and maintained in the context of the Open Perimetry Initiative, which serves as a platform for continuous and transparent development of perimetric methods in the future [23].

### 4.4. Limitations

The major limitation of our study is the small sample size. However, the study was a pilot for optimizing the research protocol. A larger study is already planned, which systematically investigates modifications of the protocol, including background luminance, stimulus size and testing locations in various retinal diseases.

The optical system of the headset introduces light losses and stray light, which may reduce effective luminance [22] as well as image contrast. Luminance calibration can mitigate, but not fully eliminate, these effects, and incorrect headset positioning may alter the perceived luminance [11].

Furthermore, it is not clear which consequence eccentric fixation has in the context of binocular fixation. The background and the fixation cross are presented to both eyes, but the stimulus itself is presented only on one side of the split screen (see Figure 2). This will also be investigated. Possibly, the alignment of the testing grid is not perfect in the HMP, because the manufacturer did not provide a Google Daydream QR code with the headset.

A further limitation of the Iowa head-mounted perimeter compared with other available HMPs is the lack of an eye tracking system that detects malfixation during the exam [10].

### 4.5. Implications for Clinicians

Head-mounted perimeters enable more widespread availability of perimetry in telemedical settings and underserved populations and offer higher patient comfort. However, they are clearly inferior to conventional perimeters in dynamic range. Therefore, they show a flattened hill of vision in normal subjects and cannot detect small losses of perimetric sensitivity in the macula, because the dimmest stimulus they can produce is too easily recognizable (ceiling effects). Furthermore, they cannot quantify large perimetric sensitivity losses (in the Iowa-HMP: sensitivities below 14 dB of Octopus sensitivity) due to floor effects. Therefore, it is not possible to differentiate poor sensitivities from absolute scotomas [22]. Furthermore, global indices like the mean defect (MD) cannot easily be compared between HMP parameters and conventional parameters, because sensitivities below the lower end of the dynamic range will affect the arithmetic average. This is an inherent limitation of the displays that are used in HMPs. Although technical developments may mitigate this limitation, the question remains whether these developments will be used in consumer electronics so that the price advantage of HMPs can be maintained.

HMPs have been validated in glaucoma and can be used there if regulatory approval is available and the clinician is aware of the limitations. However, it has not been validated in retinal disease [10]. To our knowledge, this is the first study on HMP in inherited retinal diseases, and our study clearly shows that HMP cannot characterize the entire range of pathological changes in IRDs and, thus, cannot replace conventional perimeters. Open-source perimeters enable researchers to develop new testing protocols that fully leverage the potential of head-mounted perimetry.

## 5. Conclusions

HMP can be used to identify characteristic patterns of visual field loss in inherited retinal diseases, but it cannot fully characterize sensitivities in the full range observed in inherited retinal diseases. Modifications in the testing protocol may limit comparability to established standards, but also offer the opportunity for improved protocols. Open software greatly facilitates research in this area.

## Figures and Tables

**Table 1:** Demographics and clinical data.

	Overall (N = 12)
Sex	
Female	6 (50.0%)
Male	6 (50.0%)
Age (years)	
Mean (SD)	56.3 (14.3)
Median [Min, Max]	58.0 [24.0, 73.0]
logMAR	
Mean (SD)	0.528 (0.444)
Median [Min, Max]	0.471 [0, 1.45]
Mean Defect (dB)	
Mean (SD)	14.3 (8.13)
Median [Min, Max]	13.8 [4.55, 28.5]
Missing	1 (8.3%)
Diagnosis	
normal	1 (8.3%)
OMD	2 (16.7%)
STGD	3 (25.0%)
RP	6 (50.0%)

OMD: Occult Macular Dystrophy (RP1L1 gene), STGD: Stargardt’s disease (ABCA4 gene), RP: Retinitis Pigmentosa.

**Table 2:** Parameters used by the two perimeters. The sensitivity decibel scale is a relative unit of measurement and, in perimetry, the $\Delta L_{max}$ is used by convention as the reference value. Therefore, 0 dB sensitivity is the brightest possible stimulus in a given perimeter or program. To account for these differences in $\Delta L_{max}$, 14 dB must be added to the sensitivities of the Iowa-HMP. For clarity, we do this only for plotting the fields.

Parameter	Octopus 900	Iowa-HMP	Comment
Background luminance	1.27 cd/m^2^	10 cd/m^2^	
$\Delta L_{max}$	318 cd/m^2^	12.76 cd/m^2^	Difference in sensitivity offset: 14 dB
Stimulus size	0.43deg	0.43deg	Goldman III
Stimulus duration	100 ms	100 ms	
Location pattern	10-2	10-2	68 locations, central 10deg
Strategy	Dynamic	ZEST	

**Table 3:** Contingency table showing the agreement between examinations concerning whether sensitivities are outside the dynamic range of the HMP.

	Octopus ≥ 14 dB	<14 dB
HMP ≥ 0 dB	1001	191
0 dB	55	385

## Data Availability

The data presented in this study are available on request from the corresponding author. The data are not publicly available due to protecting of the privacy of volunteers.

## Abbreviations

The following abbreviations are used in this manuscript:

IRD	inherited retinal disease
RP	retinitis pigmentosa
STGD	Stargardt’s disease (ABCA4 gene associated)
OMD	occult macular dystrophy (RP1L1 gene associated)
HMP	Head-mounted perimetry
SAP	Standard-automated perimetry
OPI	Open Perimetry Initiative
$\Delta L_{max}$	maximal luminance increment for a given perimeter
$\Delta L_{thres}$	light increment that is barely visible for the observer at a given location
Dynamic strategy	Staircase-based algorithm for threshold detection used by Ocmiddleus perimeters
ZEST	Algorithm for threshold detection: Zippy Estimation for Sequential Testing
dB	decibel
perimetric sensitivity	ability to detect a small $\Delta L_{thres}$ for a given location [dB]; 0 dB signifies inability to detect $\Delta L_{max}$ for a given perimeter
diagnostic sensitivity/specificity	ability of a diagnostic test to correctly detect presence or absence of a condition
ND filter	Neutral Density filter
IQR	Interquartile Range
